# Agglutination of peripheral blood lymphocytes from cancer patients and not from healthy controls, with the F2A1 histone fraction.

**DOI:** 10.1038/bjc.1975.130

**Published:** 1975-07

**Authors:** D. Sabolović, N. Sabolović, A. Moutte, S. Leibovici, B. Sauvezie, P. Chollet, R. Plagne

## Abstract

**Images:**


Br. J. Cancer (1975) **32**, 28

### AGGLUTINATION OF PERIPHERAL BLOOD LYMPHOCYTES FROM CANCER PATIENTS AND NOT FROM HEALTHY CONTROLS, WITH THE F2A1 HISTONE FRACTION

D. SABOLOVIĆ, N. SABOLOVIĆ, A. MOUTTE, S. LEIBOVICI, B. SAUVEZIE, P. CHOLLET AND R. PLAGNE

*From the INSERM Research Unit 95, Vandoeuvre-lès-Nancy 54500; Center for Preventive Medicine, Vandoeuvre-lès-Nancy; Institut Gustave Roussy, Villejuif; Hôpital Foch Suresne and Center Jean Perrin, Clermont-Ferrand*

Received 7 March 1975. Accepted 27 March 1975

**Summary.—A simple, rapid histone agglutination test (HAT) is described. It consists of incubation in microplates or in microtubes of blood lymphocytes isolated from cancer patients and patients with non-malignant diseases with microquantities of histone fraction F2A1. Positive reaction is shown by massive agglutination of lymphocytes of the patients tested (126 subjects): this test was positive in 76% of cases. All controls (59 subjects) were negative.**

In 1970 Field and Caspary reported that the incubation of peripheral blood lymphocytes from cancer patients with basic protein of myelin in the presence of guinea-pig macrophages lowered the electrophoretic mobility of the macrophages. This test was called the MEM (macrophage electrophoretic mobility) test and was confirmed by Pritchard *et al.* in 1972.

Acid extracts of tumours were found to be a more effective antigen in this test (Caspary and Field, 1971; Carnegie, Caspary and Field, 1973) and in 1973 Johns *et al.* reported that histone fractions isolated from calf thymus gave a positive reaction in the MEM test.

These results prompted us to see whether the reaction of lymphocytes with histone fraction could be measured directly, *i.e.* if incubation of lymphocytes from cancer patients with histone fractions changes their electrophoretic mobilities. Moreover, we wished to determine which population of blood lymphocytes (T and B) as detected by electrophoretic analysis (Sabolović, Sabolović and Dumont, 1972) reacts with the histone in the test.

The first patients studied (3 acute lymphocytic leukaemias, one chronic lymphocytic leukaemia and 5 solid tumours) showed a complete loss of cell surface charge of all their lymphocytes after 10 min incubation with histone, and the measurement of electrophoretic mobilities was quite impossible. Control lymphocytes were not affected.

This positive reaction was accompanied by a discrete microagglutination of the lymphocytes which was maximal after 30 min incubation, and this criterion is now used by us routinely to test the positive reactions. In this paper we report a preliminary observation on 126 patients with malignant and non-malignant diseases and 59 healthy controls using histone agglutination test (HAT).

### MATERIALS AND METHODS

Lymphocytes were isolated by Ficoll–Isopaque mixture as described previously (Sabolović *et al.*, 1974). About 5 ml of blood were sufficient to do this test. The lymphocyte suspension must be as pure as possible; if platelet, monocyte and granulocyte contamination are high then washing with Medium 199 and keeping the cells at room temperature for an hour promote the aggregation of platelets and the attachment of the monocytes and granulocytes to the glass. In the first series of experiments the test was done in microplates and results were evaluated microscopically. In the

Histone agglutination test in microtubes. Five min reaction. ×4.

second series the test was done in microtubes (41 × 8 mm) and evaluated macroscopically. Both techniques gave the same results.

*Test in "microtest plates".*—Lymphocytes were washed with Hanks' medium, and diluted with Hanks' medium at concentrations of 3000 lymphocytes/mm$^3$. Histone fractions (gift of Dr Johns, Chester Beatty Institute) were dissolved in 0·145 mol/l NaCl. Serial dilutions of histone (starting with 10 μg in 25 μl in the first well) were then mixed with equal volumes of lymphocytes (*i.e.* 75,000 lymphocytes in 25 μl per well). The plates were incubated for 30 min at 37°C and examined under the microscope.

*Test in microtubes.*—Lymphocytes were washed again with PBS and suspended in PBS at a concentration of 3000 lymphocytes/mm$^3$. 0·25 ml of this suspension was distributed in microtubes (41 × 8 mm) and 20 μg of histone fraction was added to the test tube. The reaction was read after 5 min and verified again after 30 min, without incubation at 37°C. Where there was a positive reaction the agglutination of lymphocytes was clearly visible (see Figure).

For electrophoretic mobility analysis, the incubation at room temperature was stopped after 5 min and cells were washed with 0·145 mol/l NaCl, pH 7·2, and measured in cylindrical electrophoresis apparatus as described (Sabolović *et al.*, 1974).

### RESULTS

Patients with various tumours were tested, together with patients with a variety of other diseases. They included patients without treatment as well as those under treatment. Moreover, some of the patients were followed up for a 5–10 month period and retested at irregular intervals. The controls included normal subjects of both sexes and all ages, normal thymus cells and tonsil lymphocytes.

Table I shows the results obtained with all patients tested and Table II shows the evolution of sensitivity to agglutination in 3 individual patients. All 5 fractions of histones (F1, F2A1, F2A2, F2B and F3) were tested but positive reactions were observed only with F2A1 and sometimes with F2A1, F2B and F3 (Table I). 76% of all patients were positive in our test compared with negative reactions in 59

TABLE I.—*Histone Agglutination Test on Cancer Patients, Patients with Non-malignant Diseases and Healthy Controls*

| Peripheral lymphocytes from patients with | Positive with | | | No. of patients tested | | |
|---|---|---|---|---|---|---|
| | F2A1 | F2B | F3 | Positive | Negative | Total |
| Cancer of ORL | + | — | — | 7 | 2 (4)‡ | 13 |
| Cancer of the lip | + | NT | NT | 1 | | 1 |
| Cancer of breast | + | — | — | 49 | 8 (2)† | 59 |
| Cancer of bladder | + | NT | NT | 1 | | 1 |
| Cancer of the prostate | + | NT | NT | | 2 | 2 |
| Retroperitoneal sarcoma | + | NT | NT | 1 | | 1 |
| Cancer of cervix | + | NT | NT | 2 | | 2 |
| Carcinoma of the colon | + | NT | NT | 2 | | 2 |
| CLL | + | — | — | 6 | 4 | 10 |
| ALL (children) | + | + | + | 6 | 1* | 7 |
| ALL (adults) | — | — | — | | 2 | 2 |
| Hodgkin's disease | + | + | — | 3 | | 3 |
| Thymoma | + | + | + | 2 | | 2 |
| Lymphoblastosarcoma | — | — | — | | 2 | 2 |
| Leukaemic lymphoblastosarcoma | + | + | + | 1 | | 1 |
| Sarcoma of the uterus | — | NT | NT | | 1 | 1 |
| Carcinoma (non-differentiated) | + | NT | NT | 1 | | 1 |
| Sezarie's disease | — | NT | NT | | 1 | 1 |
| Lupus erythematosus | + | — | — | 2 | | 2 |
| Rheumatoid arthritis | + | NT | NT | 7 | 1 | 8 |
| Connective tissue disease | + | NT | NT | 1 | | 1 |
| Sciatica | + | NT | NT | 1 | | 1 |
| Rheumatic gout | + | NT | NT | 1 | | 1 |
| Chronic arthrosis | — | — | — | | 1 | 1 |
| Coombs positive patient | + | NT | NT | 1 | | 1 |
| Graft-versus-host disease | + | NT | NT | 1 | | 1 |
| Asthma | — | NT | NT | | 1 | 1 |
| Normal tonsil | — | NT | NT | | 1 | 1 |
| Normal thymus cells | — | — | — | | 2 | 2 |
| Healthy controls | — | — | — | | 59 | 59 |

NT = not tested; * died on the same day; † benign tumours of the breast; ‡ irradiated.

Agglutination with histone fractions was performed in microplates or in tubes. Equal concentrations of lymphocytes (3000/mm$^3$) were mixed with histone fractions and the reaction was scored after 30 min of incubation at room temperature (see text).

TABLE II.—*Histone Agglutination Test on Individual Patients*

| Patient | Date | Active fraction | Dilution of histone | | | | |
|---|---|---|---|---|---|---|---|
| | | | 10 μg | 2·5 μg | 1·25 μg | 0·62 μg | 0·31 μg |
| G.D. (18 years old) | 22.1.74 | F2A1 | + | | | | |
| Acute lymphocytic leukaemia in | 5.2.74 | F2A1 | + | + | + | + | |
| remission | 12.3.74 | F2A1 | + | + | + | | |
| | 7.10.74 | F2A1 | + | + | + | + | + |
| | 15.2.75 | F2A1 | + | + | | | |
| R.A. (14 years old) | 21.1.74 | F2A1 | + | | | | |
| Acute lymphocytic leukaemia with | 22.1.74 | F2A1 | + | | | | |
| circulating lymphoblasts | 25.1.74 | F2A1 | + | | | | |
| | 18.2.74 | F2A1 | + | + | + | | |
| | 12.3.74 | F2A1 | + | + | + | | |
| | 13.5.74 | F2A1 | + | + | + | + | |
| | | F2B | + | + | + | + | |
| | | F3 | + | + | + | | |
| | Died | | | | | | |
| B.L. | 29.1.74 | F2A1 | + | + | + | | |
| Cancer of breast | 8.2.74 | F2A1 | + | + | + | | |
| | 14.2.74 | F2A1 | + | + | + | | |
| L.P.S. | 8.2.74 | F2A1 | + | | | | |
| Cancer of breast. Irradiation started | 14.2.74 | F2A1 | + | + | + | + | |

Histone agglutination test was performed in microtest plates as described in the text.

healthy controls. In the breast cancer group, for instance, positive reactions were observed in 86% of cases.

Not all patients were positive for a given group but it must be noted that some of the patients were in an advanced stage of the disease: one patient with acute lymphocytic leukaemia who was negative in HAT died on the same day and one woman with cancer of the breast, negative in HAT, had a multiple metastasis, 4 patients with cancer of ORL received local body irradiation and were negative in the test.

Benign tumours of the breast, chronic arthrosis, Sezarie's disease, cancer of the prostate and acute lymphocytic leukaemia in adults and lymphoblastosarcoma were all negative in the HAT.

Two patients with acute lymphocytic leukaemia (children) and 2 patients with cancer of the breast were followed up and retested at different periods of the disease (Table II). It can be seen that the sensitivity to histone agglutination changes during the evolution of the disease: patient R.A. became positive to the fraction F3 just before death; patient L.P.S. had an increased positivity after irradiation.

Poly-L-arginine (Biochemicals) and poly-L-lysine were tested and found to be negative even at doses of 50 $\mu g$/75,000 lymphocytes. Positive reactions were obtained with Poly-L-arginine (Sigma) in the 2 cases tested. Incubation of HAT-positive lymphocytes with PPD antigen or with sheep red cells for 15 min did not provoke agglutination and did not change their electrophoretic mobilities either.

When the reaction with the histone fraction was very strong, all cells were agglutinated and measurement of electrophoretic mobilities was impossible, but if the cell concentration was diluted six-fold and histone fraction added, no agglutination occurred at all, and then an electrophoretic analysis became possible: all lymphocytes were immobilized in the electric field and even became charged positively, *i.e.* moved in the opposite direction. These experiments also demonstrate the importance of lymphocyte concentration per $mm^3$ in order to assure agglutination in the positive cases.

### DISCUSSION

The macrophage electrophoretic mobility test (MEM) devised by Field and Caspary (1970) measures the lymphocyte–histone interaction indirectly, *i.e.* a soluble principle released by this interaction retards the electrophoretic mobilities of guinea-pig macrophages (Johns *et al.*, 1973).

Our results show that the histone–lymphocyte interaction can be measured directly using electrophoretic mobility of lymphocytes or using the histone agglutination test (HAT) which is in contrast very simple. The agglutination of the peripheral lymphocytes with F2A1 histone fraction and loss of cell surface charge (or reversal of this charge from negative to the positive one) takes a few seconds or minutes, suggesting an immediate reaction on the cell surface between sensitized lymphocytes and histone protein.

Lymphocytes from 59 normal, healthy individuals do not react in this test but 76% (128 cases) of patients with malignant and non-malignant diseases possess reactive lymphocytes in the circulation. It is not excluded that lymphocytes in normal individuals are completely devoid of " receptors " for F2A1 histone fraction but the number of these lymphocytes or concentration of such a " receptors " must be very feeble.

We focused our attention at first on the study of cancer patients and later we found that non-cancerous diseases also react and for this reason the HAT cannot be considered as specific for cancer.

Using the MEM test, Field and Caspary (1972; Field, Caspary and Smith, 1973) stated that any patient with neurological or viral disease was unsuitable for cancer testing in the MEM and also found that some inflammatory, non-malignant diseases gave a positive reaction.

What is the meaning of the lymphocyte–histone interaction? Caspary and Field (1971) and Dickinson and Caspary (1973) supported the idea that peripheral lymphocytes become sensitized to a common tumour antigen present in tumour tissue and also in all myelinated nervous tissue. Structurally related basic cancer protein, encephalitogenic protein to F2A1 histone fraction, might explain the antigen cross reactivity in the MEM test. Our interpretation follows the idea that during any pathological process (cancerous or not) involving reaction of the immune system, lymphocytes become sensitized to the self-antigens in parallel. For instance, the virus may be responsible for this deviation of immune reaction (Allison *et al.*, 1971). It is relevant to this that one Coombs' positive patient and one patient grafted with lymphoid cells and undergoing the graft-versus-host reaction were positive in the HAT.

Our test discriminates between benign and malignant tumours of the breast; the former were negative in the HAT. Negative results were observed in some patients in the ORL group treated with irradiation and in some patients in an advanced stage of the disease, as has also been reported by Field *et al.* (1973) for the MEM test. In contrast to the MEM test, we obtained positive reactions in acute lymphocytic leukaemia during relapse or remission in children and in most chronic lymphocytic leukaemia. Our findings also corroborate a recent study of Fish, Pritchard and Deeley (1974) who showed that the incubation of lymphocytes from cancer patients with F2A1 histone fraction for 90 min induces the appearance in the supernatant fluid of a component of molecular weight smaller than the original histone F2A1 fraction. They suggested that F2A1 fraction is degraded by the proteolytic action of lymphocytes from cancer patients. These experiments also indicate that an active interaction between cancer lymphocytes and F2A1 histone fraction does take place, but these authors had not tested any of the patients with non-malignant diseases.

It is clear that much work must be done in order to elucidate the mechanism of the histone–lymphocyte reaction *in vitro* as well as the pathway of the *in vivo* sensitization, and to determine whether the F2A1 reaction could be useful in the early detection of pathological process in the organism, and also its relationship with the immunological system. For this purpose, the histone agglutination test (HAT) we have described is simple, rapid and requires a minimum of laboratory practice.

This work was supported by INSERM (ATP No. 16) and by Fondation pour la Recherche Médicale Française. We are indebted to Professor J. L. Amiel and Dr J. Rouësse who allowed us to study most of the patients described here.

### REFERENCES

Allison, A. C., Denman, A. M. & Barnes, R. D. (1971) Cooperating and Controlling functions of Thymus derived Lymphocytes in Relation to Autoimmunity. *Lancet*, ii, 135.

Carnegie, P. R., Caspary, E. A. & Field, E. J. (1973) Isolation of an Antigen from Malignant Tumours. *Br. J. Cancer*, **28**, Suppl. I, 219.

Caspary, E. A. & Field, E. J. (1971) Specific Lymphocyte Sensitization in Cancer. Is there a Common Antigen in Human Malignant Neoplasia? *Br. med. J.*, ii, 613.

Dickinson, J. P. & Caspary, E. A. (1973) The Chemical Nature of Cancer Basic Protein. *Br. J. Cancer*, **28**, Suppl. I, 224.

Field, E. J. & Caspary, E. A. (1970) Lymphocyte Sensitization: an *in vitro* Test for Cancer. *Lancet*, ii, 1337.

Field, E. J. & Caspary, E. A. (1972) Spontaneous Lymphocyte Reactivity in the Presence of Virus Infection. *Lancet*, i, 963.

Field, E. J., Caspary, E. A. & Smith, K. S. (1973) Macrophage Electrophoretic Mobility (MEM) Test in Cancer: a Critical Evaluation. *Br. J. Cancer*, **28**, Suppl. I, 208.

Fish, R. G., Pritchard, J. A. V. & Deeley, T. J. (1974) Human Peripheral Lymphocytes and Cancer. *In vitro* Studies on the Basic Protein, Histone F2A1 Fraction. *Br. J. Cancer*, **30**, 222.

Johns, E. W., Pritchard, J. A. V., Moore, J. L., Sutherland, W. H., Joslin, C. A. F., Forrester,

J. A., DAVIES, A. J. S., NEVILLE, A. M. & FISH, R. G. (1973) Histone and Cancer Test. *Nature, Lond.*, **245**, 98.

PRITCHARD, J. A. V., MOORE, J. L., SUTHERLAND, W. H. & JOSLIN, C. A. F. (1972) Macrophage Electrophoretic Mobility (MEM) Test for Malignant Disease: an Independent Confirmation. *Lancet*, ii, 627.

SABOLOVIĆ, D., SABOLOVIĆ, N. & DUMONT, F. (1972) Identification of T and B Cells in Mouse and Man. *Lancet*, ii, 927.

SABOLOVIĆ, N., SABOLOVIĆ, D., DUMONT, F. & SIEST, G. (1974) Electrophoretic Mobility, Rosette-formation and Surface Immunoglobulins of Gradient Fractionated Human Blood Lymphocytes. *Biomedicine*, **21**, 86.


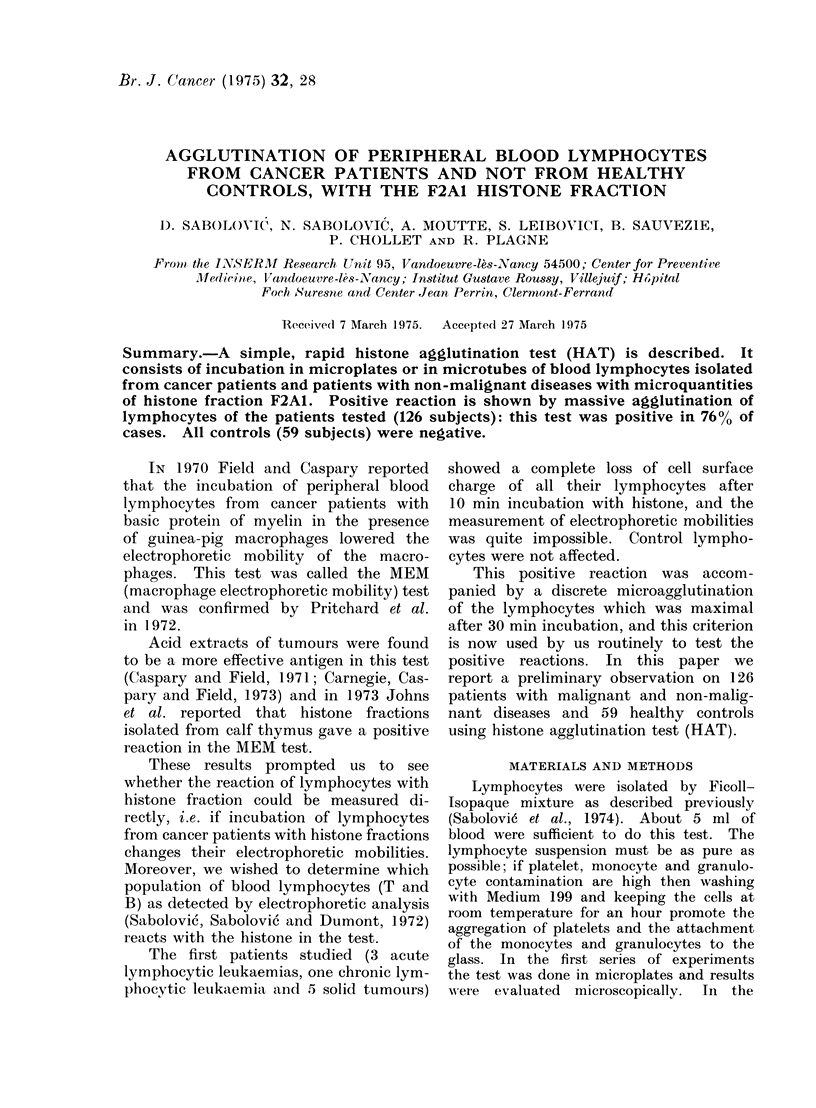

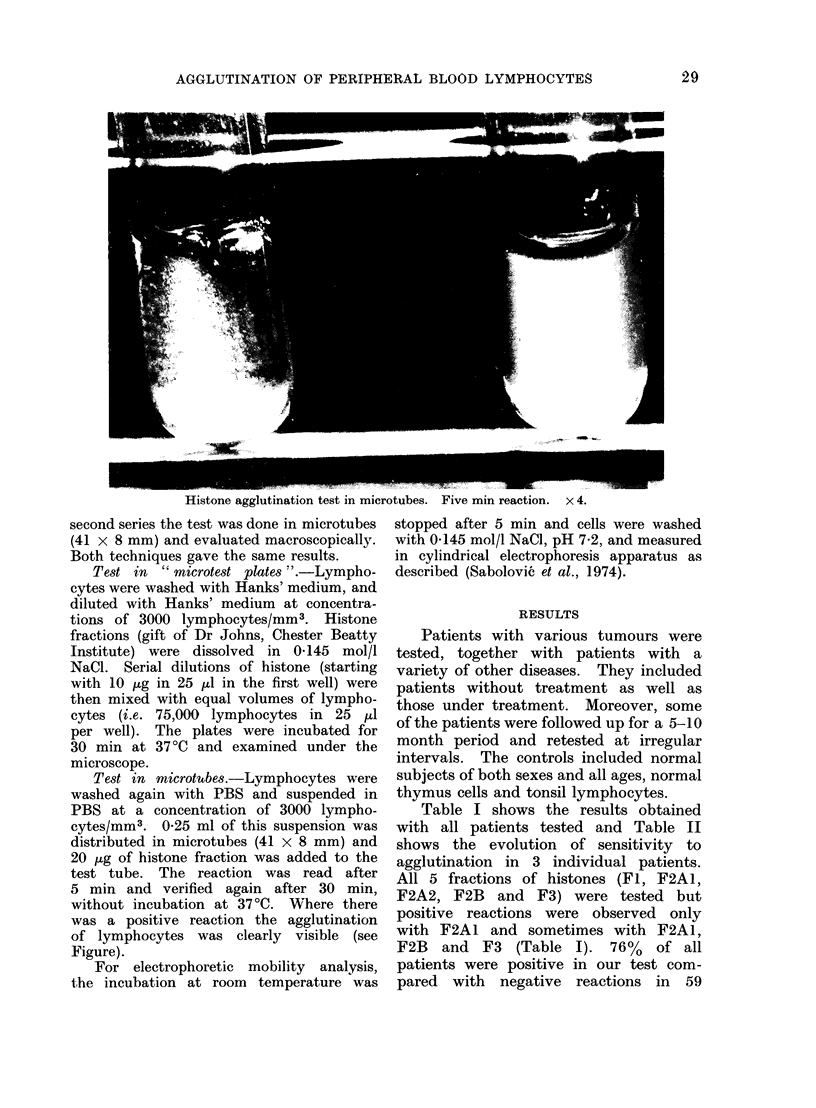

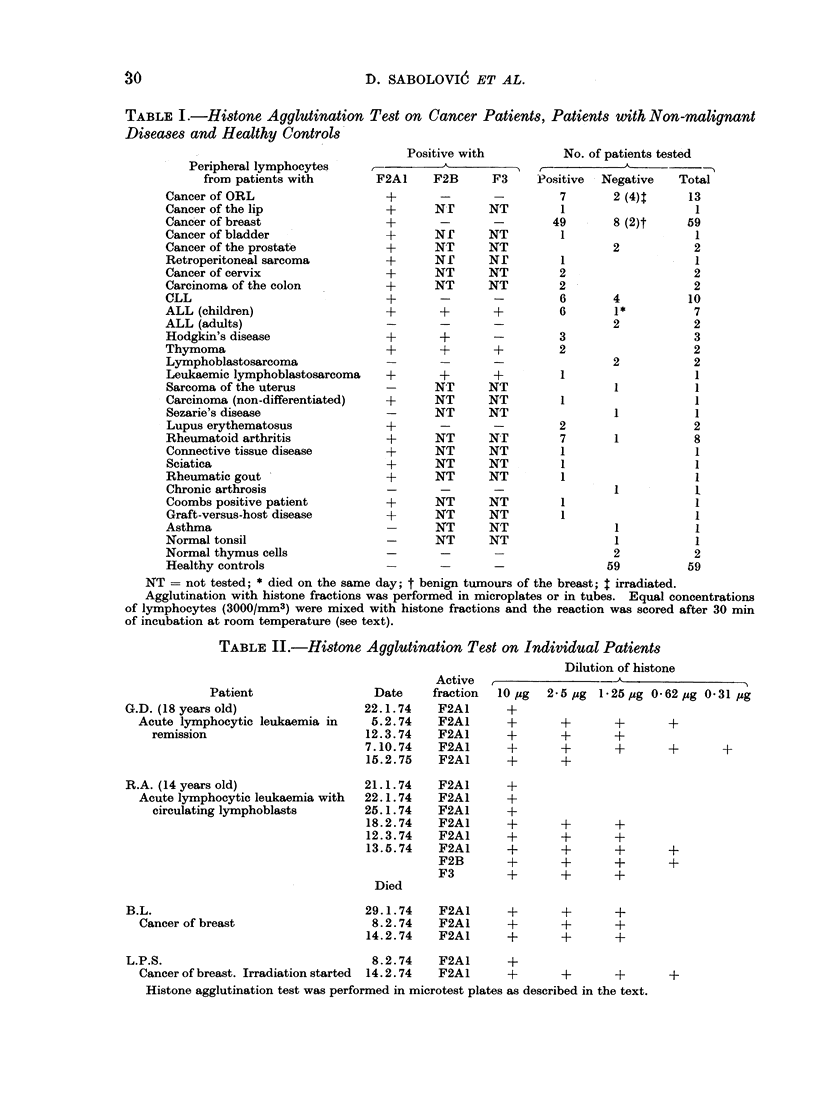

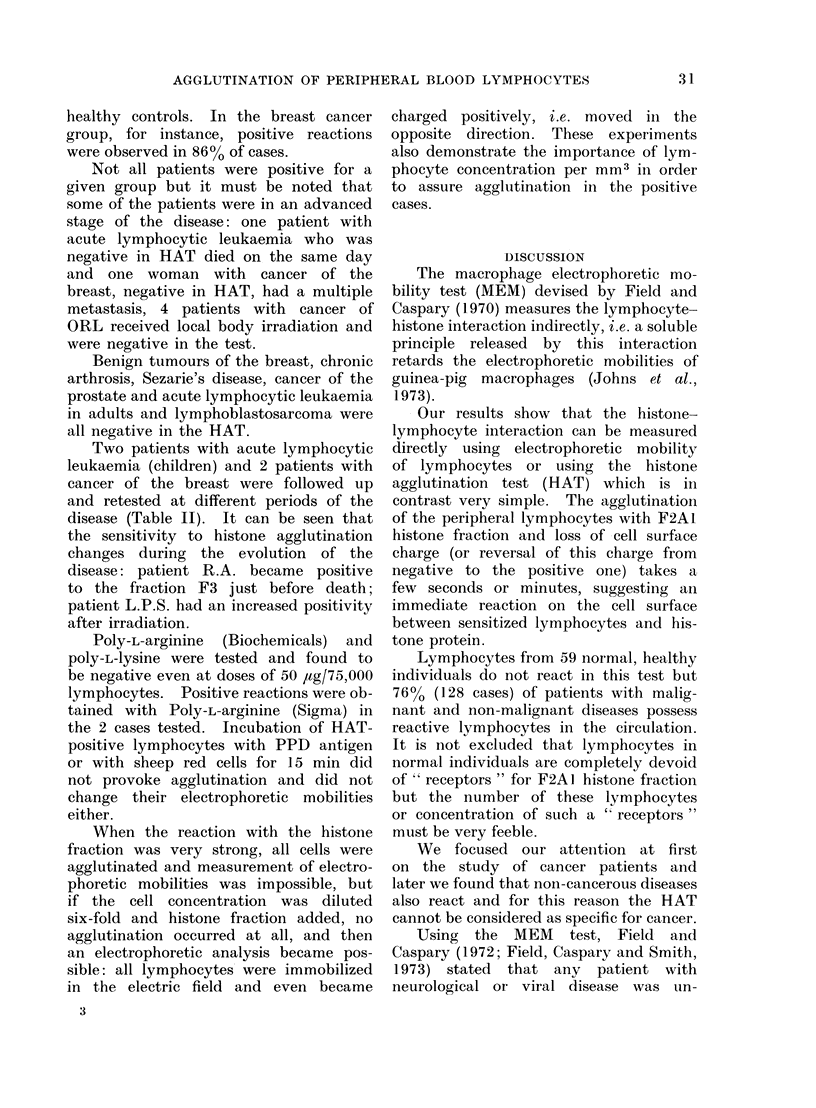

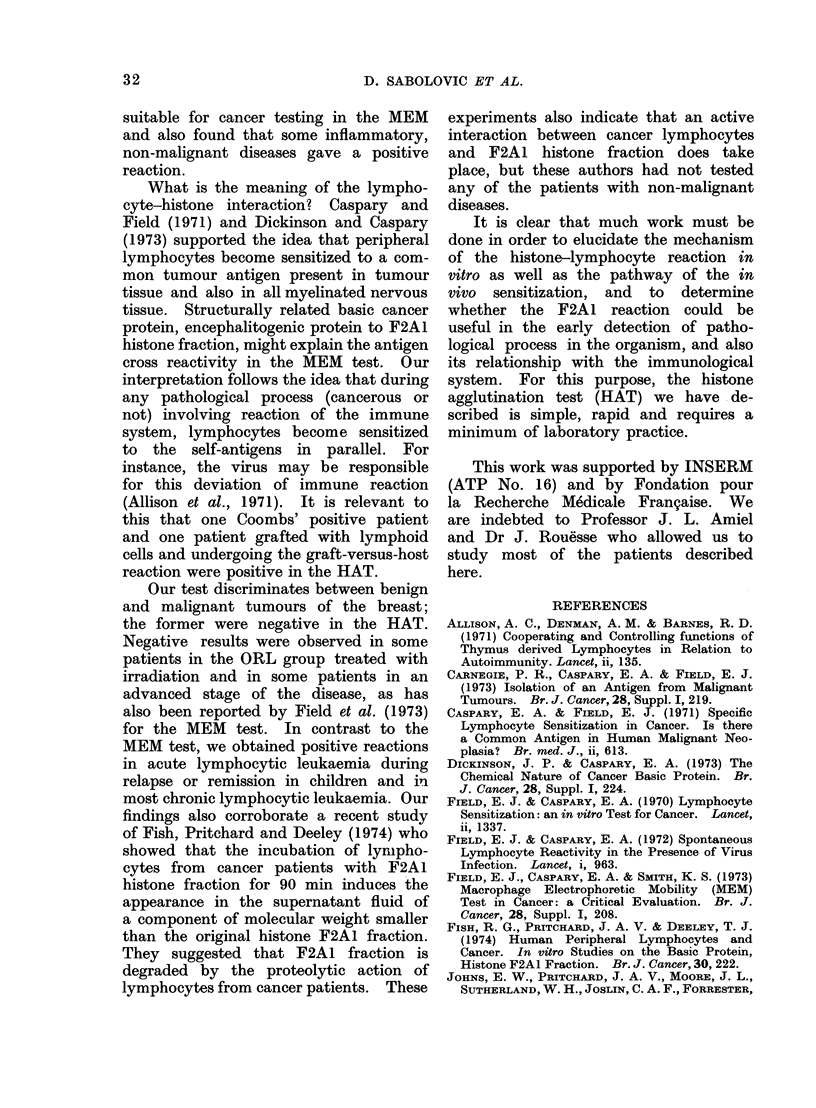

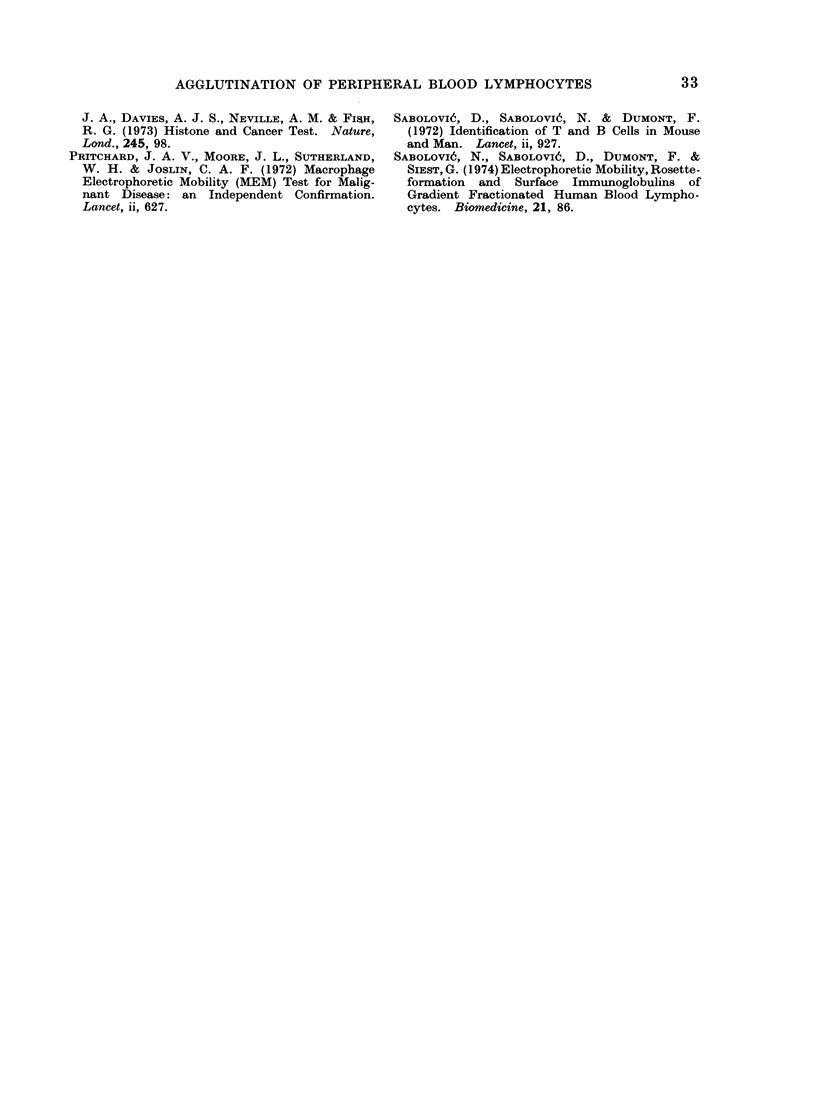


## References

[OCR_00643] Allison A. C., Denman A. M., Barnes R. D. (1971). Cooperating and controlling functions of thymus-derived lymphocytes in relation to autoimmunity.. Lancet.

[OCR_00649] Carnegie P. R., Caspary E. A., Field E. J. (1973). Isolation of an "antigen" from malignant tumours.. Br J Cancer Suppl.

[OCR_00654] Caspary E. A., Field E. J. (1971). Specific lymphocyte sensitization in cancer: is there a common antigen in human malignant neoplasia?. Br Med J.

[OCR_00660] Dickinson J. P., Caspary E. A. (1973). The chemical nature of cancer basic protein.. Br J Cancer Suppl.

[OCR_00670] Field E. J., Caspary E. A. (1972). "Spontaneous" lymphocyte reactivity in the presence of virus infection.. Lancet.

[OCR_00665] Field E. J., Caspary E. A. (1970). Lymphocyte sensitisation: an in-vitro test for cancer?. Lancet.

[OCR_00675] Field E. J., Caspary E. A., Smith K. S. (1973). Macrophage electrophoretic mobility (MEM) test in cancer: a critical evaluation.. Br J Cancer Suppl.

[OCR_00681] (1974). Human peripheral lymphocytes and cancer. In vitro studies on the basic protein, histone F2A1 fraction.. Br J Cancer.

[OCR_00697] Pritchard J. A., Moore J. L., Sutherland W. H., Joslin C. A. (1972). Macrophage-electrophoretic-mobility (M.E.M.) test for malignant disease. An independent confirmation.. Lancet.

[OCR_00711] Sabolović N., Sabolović D., Dumont F., Siest G. (1974). Electrophoretic mobility, rosette formation and surface immunoglobulins of gradient fractionated human blood lymphocytes.. Biomedicine.

